# What Lies Beyond the Thyroid? A Case of Neck Pain, Fever, and Hypertension

**DOI:** 10.1002/jgf2.70119

**Published:** 2026-04-24

**Authors:** Atsuhiko Sunaga, Takuya Inoue, Atsushi Omoto

**Affiliations:** ^1^ Department of Rheumatology Matsushita Memorial Hospital Osaka Japan; ^2^ Department of General Internal Medicine Japanese Red Cross Kyoto Daiichi Hospital Kyoto Japan

A 40‐year‐old Japanese man presented with a 6 month history of fever, weight loss, neck tenderness and swelling, tachycardia, and refractory hypertension. Despite treatment with bisoprolol and amlodipine, the blood pressure of the patient remained above 160/110 mmHg. The patient was referred to our hospital with suspected subacute thyroiditis. However, the 6 month duration of symptoms was atypical for this condition. Physical examination revealed bilateral neck pain without thyroid tenderness, and vascular bruits were not detected. Thyroid ultrasonography revealed a normal thyroid gland. Given the anatomical location of the tenderness near the carotid arteries, carotid ultrasonography was performed at the same time, revealing significant bilateral thickening of the carotid artery wall consistent with the “macaroni sign,” which presents smooth, homogeneous circumferential wall thickening (Figure 1). Laboratory tests revealed leukocytosis (11,500/μL), elevated C‐reactive protein levels (5.13 mg/dL), and an increased erythrocyte sedimentation rate (51 mm/h). Supplementary tests for secondary hypertension, including thyroid function (thyroid‐stimulating hormone, 1.52 μU/mL; free T3, 3.00 pg/mL; free T4, 1.18 ng/mL), plasma renin activity (1.3 ng/mL/h), serum aldosterone (53.3 pg/mL), and morning cortisol (18.1 μg/dL) were within the normal ranges. Contrast‐enhanced computed tomography (CT) revealed the “double‐ring sign” in both the carotid arteries (Figure 2A) and the abdominal aorta (Figure 2B), characterized by a well‐enhanced outer ring suggesting adventitial inflammation and a poorly enhanced inner ring representing intimal edema, consistent with large‐vessel vasculitis [1]. Additionally, the patient tested positive for HLA‐B52. Based on these findings, a diagnosis of Takayasu arteritis (TA) was established.

**FIGURE 1:**
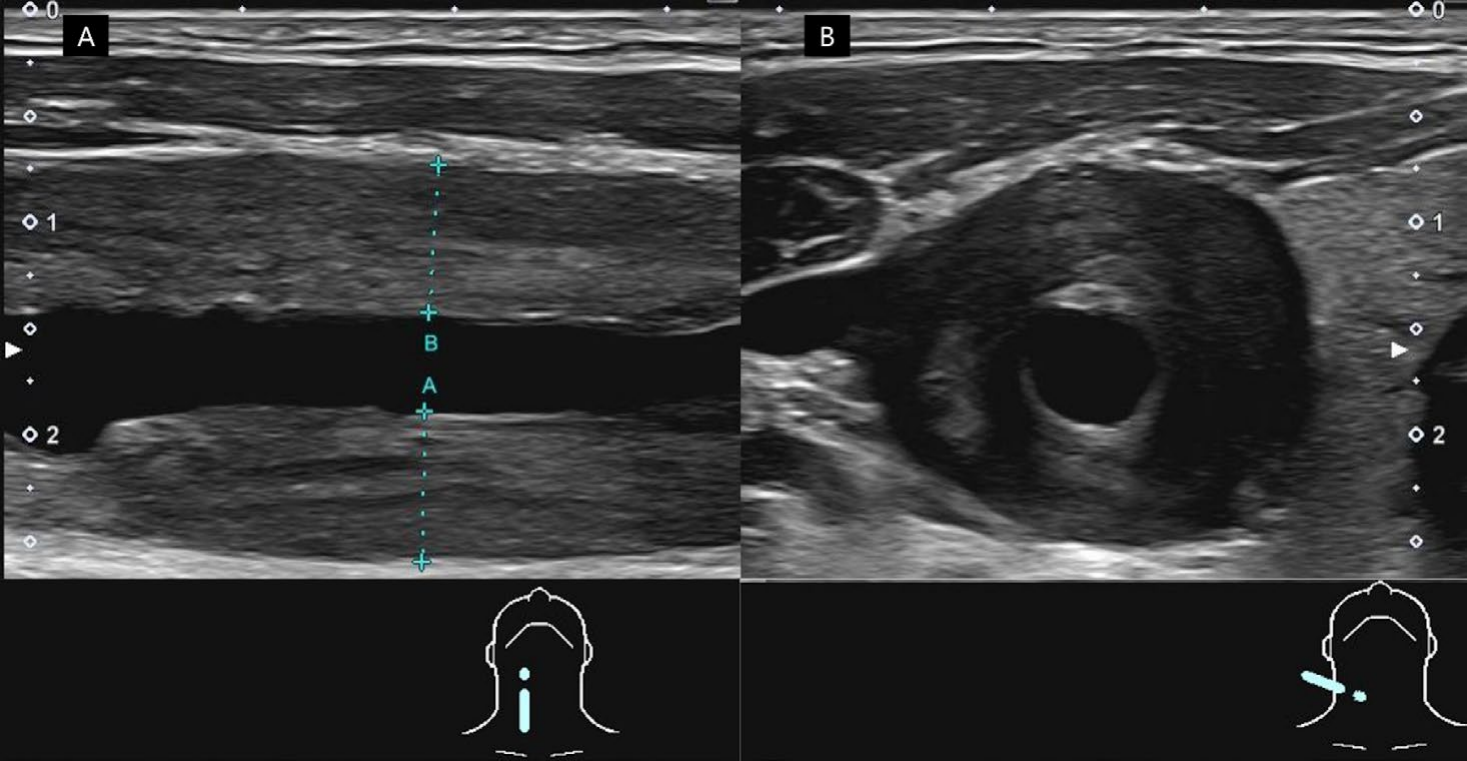
Carotid ultrasonography of the right common carotid artery. (A) Longitudinal view reveals smooth, homogeneous wall thickening. (B) Transverse view reveals concentric circumferential thickening, consistent with the “macaroni sign”.

**FIGURE 2:**
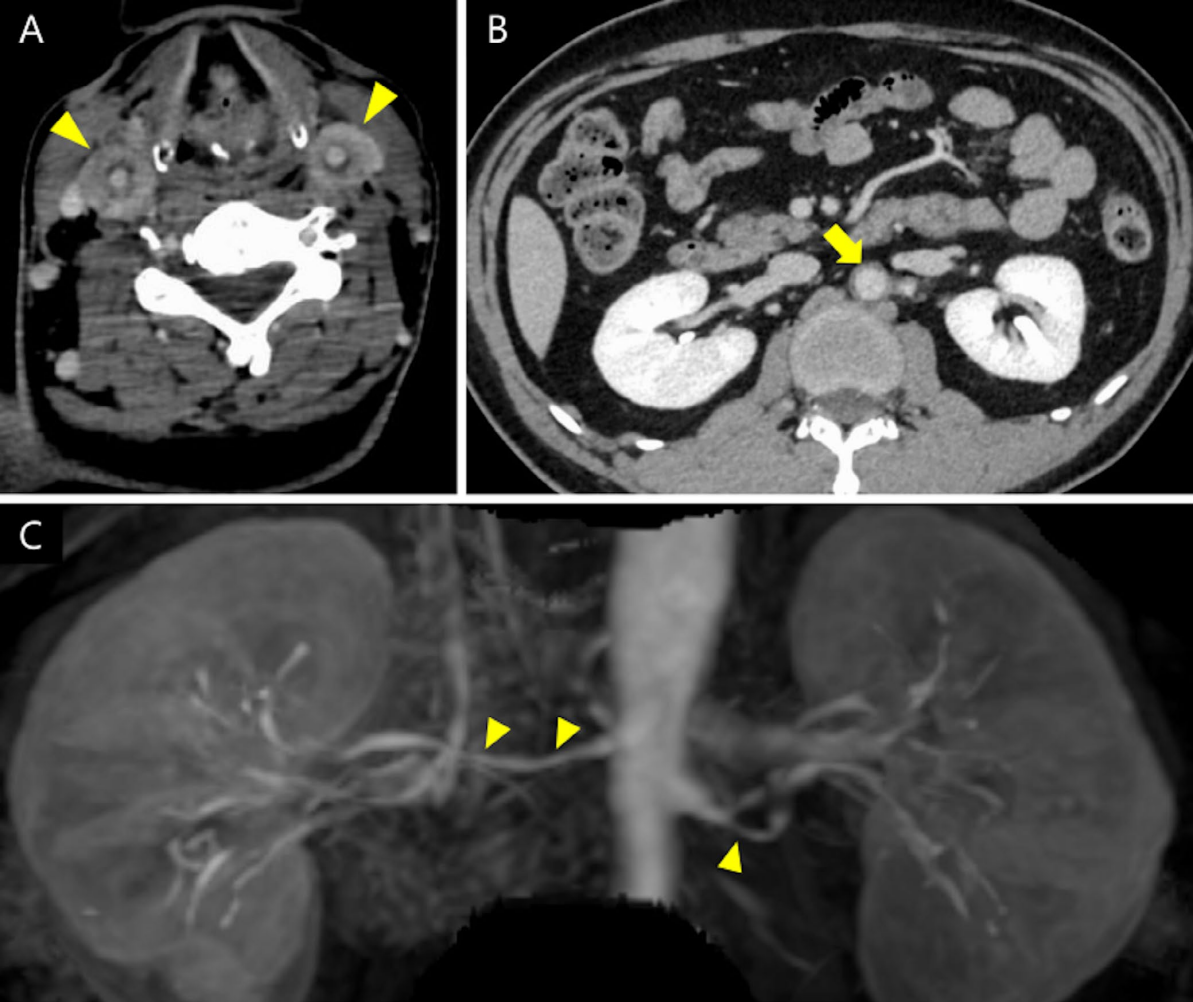
Contrast‐enhanced CT and MR imaging. (A, B) Contrast‐enhanced CT shows wall thickening with enhancement in the carotid arteries (A, yellow arrowhead) and abdominal aorta (B, yellow arrow). (C) MR angiography revealed diffuse stenosis of the bilateral renal arteries (yellow arrowhead). CT, computed tomography; MR, magnetic resonance.

Although abdominal bruits were not detected, magnetic resonance (MR) angiography revealed bilateral renal artery stenosis (Figure 2C) consistent with TA, and the etiology of the patient's persistent hypertension was attributed to renovascular pathology. Coronary angiography revealed coronary artery stenosis, indicating TA cardiac involvement. Transthoracic echocardiography revealed diffuse left ventricular hypokinesis, attributed to hypertension and coronary ischemia. The patient was initially treated with high‐dose prednisolone and methotrexate. Treatment was followed by adalimumab owing to a flare‐up during tapering, which improved both neck pain and blood pressure.

TA is a form of large‐vessel vasculitis that predominantly affects young Asian individuals and can lead to various vascular complications such as extremity claudication, aortic regurgitation, stroke/transient ischemic attack, coronary artery disease, and renovascular hypertension [2]. Delaying TA diagnosis may result in the progression of vascular lesions, worse long‐term vascular outcomes, and even death [3]. As observed in this case, the initial symptoms of TA are often nonspecific and can mimic those of thyroiditis. However, careful history taking, physical examination, and carotid ultrasonography can help distinguish them. When young patients present with neck pain, fever, systemic symptoms, and refractory hypertension, clinicians should not focus solely on thyroid disease; TA should be considered, and early carotid ultrasonography can be key to establishing the diagnosis.

## Author Contributions

**Atsushi Omoto:** writing – review and editing. **Atsuhiko Sunaga:** conceptualization, writing – original draft, data curation, investigation, visualization. **Takuya Inoue:** investigation, conceptualization, writing – review and editing.

## Funding

No specific grants were received from any funding agency in the public, commercial, or nonprofit sectors.

## Ethics Statement

Ethics approval was waived by the institutional review board because of the design of this study.

## Consent

Written informed consent was obtained from the patient for the publication of this case report and the accompanying images.

## Conflicts of Interest

A.S. received honoraria for lectures from Abbvie. A.O. received honoraria for lectures from AbbVie, Chugai, Eisai, Ono, Daiichi Sankyo, Astellas, AstraZeneca, Mitsubishi Tanabe Pharma, Pfizer, Asahi Kasei Pharma, Novartis, Gilead, Janssen, GSK, and Eli Lilly. T.I. has no conflicts of interest to declare.

## Data Availability

Data sharing not applicable to this article as no datasets were generated or analysed during the current study.
